# Specificity models in MAPK cascade signaling

**DOI:** 10.1002/2211-5463.13619

**Published:** 2023-06-11

**Authors:** Yan Ma, Jade Nicolet

**Affiliations:** ^1^ Department of Plant Molecular Biology, Biophore, UNIL‐Sorge University of Lausanne Switzerland

**Keywords:** MAPK cascade, plant signaling, RLK receptors, signaling specificity

## Abstract

The precise execution of various cellular functions relies on the maintenance of signaling specificity from input detection to cellular outputs. However, diverse signaling pathways share similar or identical intermediate components. A well‐conserved intermediate, the Mitogen‐Activated Protein Kinase (MAPK) cascade, participates in a myriad of signaling pathways, regulating signal transduction from input to output. This typifies the “hourglass conundrum”, where a multitude of inputs and outputs all operate through a limited number of common intermediates. Therefore, understanding how MAPK cascades regulate a variety of outputs with specificity is a fundamental question in biology. This review highlights four major insulating mechanisms that improve signaling specificity: selective activation, compartmentalization, combinatorial signaling, and cross‐pathway inhibition. We focus on plant pathways that share MAPK cascade components and compare mechanisms with those of animals and yeast. We hope this conceptual overview will aid future studies to better understand plant signaling specificity.

## Graphical abstract

Diverse signaling pathways share similar or identical intermediate components. The MAPK cascade is a well‐conserved intermediate, participating in myriad signaling pathways. The precise execution of various cellular functions relies on signaling specificity from input detection to cellular outputs. Comparing plant and animal pathways, this review highlights four major insulating mechanisms that improve signaling specificity.

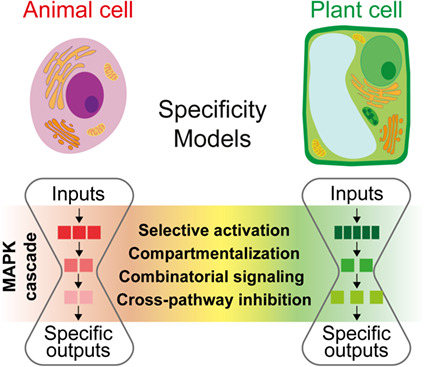

**Abbreviations**
Please refer to Glossary section at the end of the article 

The ability to respond to external stimuli is a fundamental characteristic of all living organisms. The smallest response unit, a cell, converts external signals into requisite cellular responses by activating appropriate signaling pathways. The evolution of multicellularity in eukaryotes followed by functional diversification of the cells has drastically increased the complexity and intricacy of signaling pathways. The execution of a myriad of cellular functions thus relies on these pathways to maintain specificity from signal input to cellular output. These outputs range from basic growth and proliferation to highly specialized functions such as development of xylem tissues for structural support and transport in vascular plants, and the adaptive immune response to a specific pathogen in mammals.

However, it is a widespread phenomenon for diverse signaling pathways to share similar or identical intermediate components. One key intermediate module, the Mitogen‐Activated Protein Kinase (MAPK) cascade, is activated by a wealth of known stimuli in plants, animals, and fungi [1, 2, 3, 4]. This typifies the “hourglass conundrum”, where a multitude of inputs each need to elicit a distinct output via a limited number of common intermediates (Fig. 1A,B **)**. The resulting high interconnectivity of signaling pathways raises an important challenge to understand how output specificity is maintained, and more precisely: *How do common signaling modules regulate a variety of outputs?* Relative specificity is achieved when one pathway's input can activate more of its own output than another pathway's output. Absolute specificity means one pathway can only activate its own output and cannot activate another pathway.

**Fig. 1:**
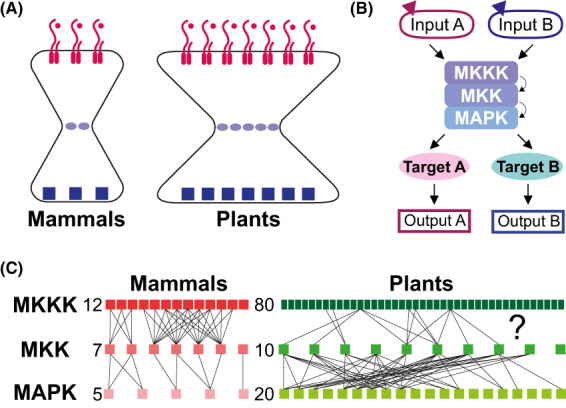
MAPK cascade as a nexus for signaling pathways. (A) Schematics of “Hourglass conundrum” in mammals and plants. A limited number of signaling intermediates (mauve oval shapes) participate in diverse signaling cascades, elicited by different inputs (exemplified by receptor complexes and ligands) and producing different outputs (blue squares). (B) A simple specificity model where two pathways share the same MAPK cascade yet can transduce different signals to produce specific outputs. (C) Schematic representation of MAPK cascade in mammals and plants. Boxes represent MKKKs, MKKs, MAPKs in humans (Red) and in *Arabidopsis thaliana* (Green) with total numbers noted on the left. Lines connecting different tiers represent major functional interactions characterized in mammals [17] and in *Arabidopsis* [18]. Note that some of these interactions are based upon *in vitro* experiments. Many of the MKKK‐MKK connections are still unknown in plants; here we illustrate only the ones mentioned in this review (MEKK1, YDA, ANP2, ANP3).

Likely as an adaptation to their sessile lifestyle, land plants have dramatically expanded their receptor kinase repertoire [5, 6]. For example, there are over 600 Receptor Like Kinase (RLK)/Pelle receptors in Arabidopsis, dwarfing the four members of the same family in humans [6]. Functional receptors detect exogenous signals to sense the external environment and detect endogenous signals to communicate with other cells [7, 8]. Plants have a de‐centralized organization, meaning that they distribute decision making and signal integration to local organs and cells. Long‐distance communication between different plant organs often operates via mobile ligands or hormones [9, 10]. Fittingly, plant genomes encode a staggering number of small secretory peptides as ligands for cell‐to‐cell communication [11]. This, together with fewer cell types than most metazoans, suggests that any single plant cell needs to incorporate more independent signals. Therefore, plants should have a more pronounced hourglass problem than their metazoan counterparts (Fig. 1A **)**, making it important to understand the mechanisms whereby undesirable crosstalk is avoided and signaling specificity maintained.

The MAPK cascades are universal signaling modules present in all eukaryotes. They serve as the nexus of diverse signaling pathways, regulating fundamental aspects of biology. A typical cascade comprises three tiers of sequentially activating kinases: MAPK kinase kinase (MKKK), MAPK kinases (MKK), and the terminal MAPK (Fig. 1B). Plants possess enlarged kinase families from all three tiers compared to other eukaryotes, albeit to a lesser degree than the expansion of RLK receptors [5, 12]. There are 80 MKKKs, 10 MKKs, and 20 MAPKs in Arabidopsis [13, 14, 15]. In contrast to the more linear cascade in yeast and mammals [16, 17], plant MAPK cascades form a web‐like network with more members present at a single tier (Fig. 1C) [18]. The expansion in plants amplifies the potential for these kinases to form many more MPKKK‐MKK‐MAPK combinations, which could enable a diversifying network perfectly suited for transmitting distinct signals. Despite their importance, only a few cascades have been characterized with all three tiers [19, 20, 21]. Moreover, only limited members in each tier were investigated, meaning that we currently only have a fragmented view of the full network. Global analyses of MKK‐MAPK interaction specificity, selective activation, and MAPK substrates have improved our overview of the full network [22, 23, 24, 25], with the caveat that validation in plants is limited and often lacks the critical cellular resolution. To date, how specificity is controlled among these MAPK cascades in plants is still not well understood.

This review highlights major insulating mechanisms that improve signaling specificity, including *selective activation, compartmentalization, combinatorial signaling, and cross‐pathway inhibition*. We focus on plant signaling pathways that share MAPK cascade components and compare them with those of animals and yeast upon which specificity models were built [26]. We also discuss various fine‐tuning mechanisms and the challenge of distinguishing desirable crosstalk versus undesirable leakage of signaling in plants. We hope our conceptual overview will aid future studies to better understand signaling specificity in plants.

## Selective activation via docking interactions

**Fig. 2:**
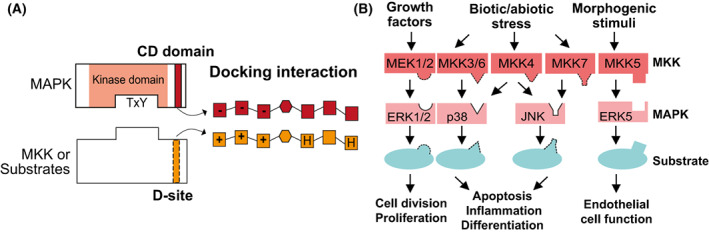
Selective activation via docking interactions. (A) Docking interactions between the MAPK and their substrates or with their upstream activators (MKKs). MAPK activation relies on the upstream MKK to phosphorylate the conserved TxY motif in the MAPK kinase domain. Solid lines represent the D‐site while dotted lines represent the CD domain. On the right, docking interaction is detailed with D‐site and CD domain properties, where positively, negatively charged and hydrophobic amino acids are represented by “+”, “−”, and “H”, respectively. The hexagon represents a 1–6 aa linker. (B) Human MAPK cascade illustrating the level of pathway specificity. See glossary “Mammalian MAPK families” for more details. Solid lines represent the D‐sites and dotted lines represent the CD‐domains.

Binding through complementary protein regions in a “lock‐and‐key” fashion is a fundamental mechanism to provide specificity. Docking interactions between the C‐terminal Docking (CD) domain in MAPKs and the docking sites (D‐sites) in MAPK substrates and regulators provide selectivity for signaling transmission (Fig. 2A) [27]. The typical D‐site motifs comprise two or three basic residues, a spacer and two hydrophobic residues separated by one other residue [27]. Positively charged D‐sites interact with complementary CD domains in MAPKs, mostly composed of negatively charged residues [27].

In mammals, most substrates recruit their cognate MAPKs via well‐characterized D‐sites [28]. The docking interactions are important to increase the local concentration of the MAPKs and thereby the phosphorylation efficiency of their substrates. For example, mutation or deletion of the D‐site in the substrate c‐Jun significantly reduces its phosphorylation by human JNK2^MAPK^ [29]. Three of the four major mammalian MAPK families (ERK1/2, p38, JNK) preferentially bind to their respective D‐sites (Fig. 2B), while noncanonical docking sites and promiscuous D‐sites exist [28, 30, 31]. Garai *et al*. [32] showed that variable residues in the D‐sites mostly contribute to the specific interaction with MAPK CD domains, and by modulating these residues, they can affect binding specificity. MKKs, the upstream activators of MAPKs, also contain D‐sites at their N termini [30]. Mammalian MAPKs generally bind better to the D‐sites of their cognate MKKs than to others, and *vice versa*, indicating that these docking interactions provide intrinsic selectivity at MKK‐MAPK level (Fig. 2B) [30]. For example, MEK1/2^MKKs^ only activate ERK1/2^MAPKs^ from their own pathway, but do not activate JNK^MAPKs^ or p38^MAPKs^ [30].

Most plant MAPKs also contain a CD domain and 7 out of the 10 MKKs in Arabidopsis carry a D‐site, with the same consensus sequence as in mammals [13]. In contrast to the high MKK‐MAPK specificity in mammals, protein microarrays revealed that a single plant MKK preferentially activating multiple MAPKs is a rule rather than an exception [23], highlighting ample potential for cross‐pathway activation of the same MAPKs. Though some of these *in vitro* interactions [20] have been confirmed in planta, many others still await validation. Nevertheless, specificity exists at MKK‐MAPK level in plants, though the degree to which docking interactions contribute to specificity is unknown. For example, AtMPK8 is activated by AtMKK3 but not by AtMKK4 upon mechanical wounding [33]. Since full AtMPK8 activation requires both AtMKK3 and the direct binding of calcium–calmodulin proteins (CaM) [33], other binding partners likely assist MKK‐MAPK specificity. Furthermore, strong affinity might not be necessary for plant MKKs to activate MAPKs, given that MAPKs are reported to be substrates of the MKKs even when they do not physically interact in a yeast two‐hybrid assay [22]. In line with this, mutation in the CD domain of the mammalian ERK2^MAPK^, or modification of the D‐site in MEK2^MKK^ does not alter their specific interaction [27, 34], indicating alternative specificity mechanisms.

Typical D‐sites as in mammalian MAPK substrates have rarely been found in plants. Given that MAPKs are known to simultaneously influence a variety of cellular processes, it seems unlikely that diverse MAPK substrates use the same motif to convey interaction specificity. In addition, the entire subgroup D of plant MAPKs lack a CD domain; therefore, an alternative mechanism must assist interactions with their substrates. Putarjunan *et al*. [35] found that AtMPK6 requires both a D‐site and a unique KRAAM motif to bind to its target (SCRM), but AtMPK3 only requires the KRAAM motif and not the D‐site. These examples demonstrate that interactions via canonical docking sites are not sufficient to explain the specificities in plant MAPK signaling. There is potential to find unique modes of action for different plant MAPKs.

## Selective activation via scaffold proteins

**Fig. 3:**
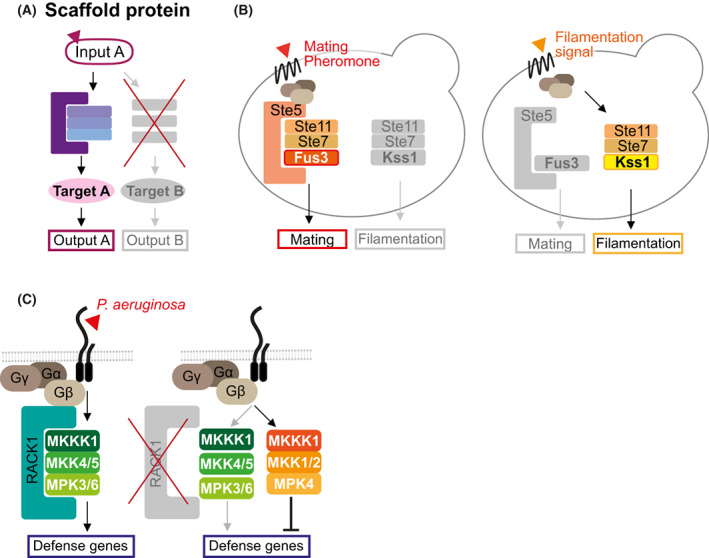
Selective activation via scaffold proteins. (A) A scaffold protein enhances specificity by selectively binding to MAPK cascade components. These kinases are prevented to be activated by another cascade which does not activate the scaffold as long as their movement on and off the scaffold is limited. (B) The yeast Ste5 scaffold protein enhances specificity of filamentation and mating signaling. Ste5 is specifically recruited during mating pheromone perception and is not recruited during filamentation. Though the two pathways share the same Ste11^MKKK^ and Ste7^MKK^, Fus3^MAPK^ activation requires Ste5, while the Kss1^MAPK^ does not. Brown ovals represent the G protein subunits: Gα, Gβ, and Gγ. (C) Upon perception of the *P. aeruginosa* pathogen protease, the Arabidopsis scaffold RACK1 binds to all the MKK1‐MKK4/5‐MPK3/6 cascade components, thereby activating defense gene expression. In the absence of RACK1 activation, MKKK1 may favor activating MKK1/2‐MPK4 to suppress immunity.

Scaffold proteins assemble consecutive signaling components into a complex to accelerate the rate of reaction and thus enhance activation [26]. By tethering selected components of one pathway while excluding others, lowering or preventing their activation, scaffolds can increase the specificity for pathways that share components (Fig. 3A). For example, the yeast scaffold protein Ste5 is specifically recruited upon mating pheromone perception to bind to all three tiers of the cascade: Ste11^MKKK^, Ste7^MKK^, and Fus3^MAPK^ (Fig. 3B) [36, 37, 38]. The resulting specific activation of Fus3^MAPK^ ensures mating outputs, which is insulated from the Ste11^MKKK^‐Ste7^MKK^‐Kss1^MAPK^ cascade activated during filamentous invasive growth (Fig. 3B) [37, 38]. Mammalian scaffold KSR1 directly interacts with Raf‐1^MKKK^, MEK1/2^MKK^, and ERK^MAPK^ to facilitate activation of the ERK pathway, important for transmitting various developmental signals [39].

Scaffolding mechanisms are widely used in yeast and mammals, yet very few plant scaffolds have been identified that sequester all three tiers of the MAPK cascade, and no homologs of Ste5 have been found in plants [18, 40]. The first example of a plant scaffold is Arabidopsis RACK1 that interacts with heterotrimeric G‐protein subunit Gβ as well as all members of the MEKK1^MKKK^‐MKK4/5‐MPK3/6 cascade upon perception of the pathogen *Pseudomonas aeruginosa* to activate immune response [41] (Fig. 3C). As MEKK1 can also activate the MKK1/2‐MPK4 cascade in negative regulation of plant immunity [42], RACK1 could be important to direct MEKK1 signaling towards MKK4/5 and prevent it from activating MKK1/2 (Fig. 3C). The structure of Arabidopsis RACK1 reveals surfaces for multiple simultaneous protein–protein interactions, indicating its potential to mediate diverse signaling [43]. RACK1's function as a multi‐signaling scaffold protein is conserved in many eukaryotes. It is considered a Gβ subunit homolog, containing the WD40 repeat domain that facilitates interactions with various proteins [43]. This domain allows the plant Gβ (AGB1) itself to interact with all 5 components of the YDA^MKKK^‐MKK4/5‐MPK3/6 cascade for embryo development [44]. As a scaffold, AGB1 assembles a specific signaling complex near the plasma membrane, potentially preventing “leakage” of YDA into other MAPK pathways [45, 46]. AGB1 can promote kinase activity, as was shown for ZAR1 (Zygotic arrest 1) [47], so it could also promote kinase activity throughout the cascade.

Substrates themselves may function as scaffolds. For example, the *Arabidopsis* protein BASL is a substrate of MPK3/6, and phosphorylated BASL recruits YDA^MKKK^ and MPK3/6 to the cell cortex, spatially concentrating signaling activity asymmetrically to create polarity [48]. In turn, the activated MPK3/6 further phosphorylates BASL, forming a positive feedback loop that eventually establishes asymmetric cell fate of the stomatal lineage [48]. MYB44 is also an MPK3 substrate in Arabidopsis that can directly interact with both MPK3 and MKK4 in the nucleus [49]. *MYB44* is an early stress‐responsive gene that is transcriptionally regulated by a MPK3‐targeted transcription factor VIP1 [50]. Though the nature of the MYB44 scaffolding benefit is unclear, we could speculate that it increases MKK4‐MPK3 signaling to promote MYB44 activation. The role of scaffolds in fine‐tuning signal transmission will be discussed later.

## Compartmentalization (tissue and subcellular localization)

**Fig. 4:**
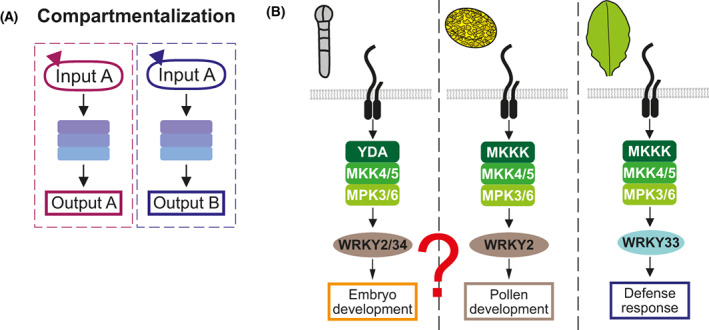
Compartmentalization. (A) The same cascade can produce different outputs when certain elements are expressed in specific tissue or subcellular compartments. Burgundy and blue boxes represent such distinct compartments. (B) MKK4/5‐MPK3/6 is involved in pollen development, embryo development, and defense response. Differential expressions of the MAPK substrates, the WRKY transcription factors, may explain some of these specificities. However, as WRKY2 is expressed in both embryo and pollen, other mechanisms may contribute to these different outputs.

Compartmentalization provides signal insulation to pathways with shared signaling components by restricting the accessibility of component(s) to specific tissues or subcellular localizations (Fig. 4A). For both plants and animals, the majority of MAPKs localize to the nucleus and/or cytosol, consistent with their targets being predominantly transcription factors [40, 51]. Nuclear translocation of MAPKs upon activation has been well documented in animals, especially for the mammalian ERK^MAPK^ family [52, 53]. The regulation of nuclear translocation could determine whether a fresh active pool of MAPKs is in the same compartment as their substrates, thus control signaling outputs [52]. How dynamic nucleocytoplasmic shuttling contributes to signaling specificity has been reviewed in plants [40, 54] and is not elaborated here.

With immobile cells and well‐defined tissue organization in plants, substrate compartmentalization in different tissues could contribute to signaling specificity. The same *Arabidopsis* MKK4/5‐MPK3/6 cascade can regulate distinct biological processes by phosphorylating different MAPK substrates in different tissues [15]. Many MAPK substrates are WRKY proteins [23], a large family of plant‐specific transcription factors regulating numerous processes [55, 56]. MKK4/5‐MPK3/6 activates WRKY34 and possibly WRKY2 for pollen development and viability [57], but activates WRKY33 for immune response upon detection of pathogens [58]. WRKY34 is strongly expressed in the pollen [59], where it activates the expression of *GPT1*, a gene important for lipid body accumulation during pollen development [60]. WRKY33 is mainly expressed in vegetative tissues and is necessary for the biosynthesis of the defense compound camalexin [58, 61]. Differential tissue availabilities of WRKY34 and WRKY33 allow the MKK4/5‐MPK3/6 cascade to activate different outputs in different tissues (Fig. 4B). However, WRKY2 is also expressed in the embryo, where it is phosphorylated by YDA^MKKK^‐MKK4/5‐MPK3 to upregulate genes important for embryogenesis [62, 63, 64]. This means that WRKY2 has disparate functions in embryo and pollen, albeit being regulated by the same MAPK cascade members. Mechanisms other than tissue compartmentalization may help to ensure differential outputs from the same WRKY2 activation pathways (Fig. 4B).

Clustering of plant MAPK cascade genes from all tiers by expression similarities broadly reflects tissue‐specific patterns [65]. These “co‐expressions maps” provide us clues where different components of the cascade could work together in a network. Nevertheless, tissue specificity of plant MAPKs is rare as the majority are broadly expressed throughout the plant [66]. Some MKKs and MKKKs are known to display preferential tissue expression. For example, AtMKK6 transcript level is higher in shoot apices and flowers where more cell division occurs, compared to mature leaves [67]. MKK6 is part of the ANP2/3^MKKK^‐MKK6‐MPK4 cascade that controls cytokinesis in Arabidopsis [67, 68]. This localization pattern of MKK6 ensures higher activity of this MAPK cascade in the dividing zones where regulation of cytokinesis is most important.

Different subcellular localization of MAPK pathway elements can provide specificity. As the example above, both MKK6 and its downstream MPK4 and MPK6 accumulate at the equatorial plane of the phragmoplast—a microtubule structure formed between the two daughter cells before division [68, 69]. So, this specific subcellular localization may enhance specificity by lowering irrelevant activations of MPK4 or MPK6 by other MAPK cascades members usually localized to nucleocytoplasmic compartments. Similarly, MKK7/9 and MKK4/5 are both activated by upstream YDA^MKKK^, and they activate MPK3/6, but show distinct activities during different stomatal development stages. Subcellular localization of autoactive MKK7/9, but not MKK4/5, to the mitochondria determines the former's ability to promote stomatal clustering during the FAMA stage [70]. Interestingly, the D‐sites of these MKKs strongly influence their ability to localize to mitochondria, as demonstrated by experiments removing/swapping the D‐sites [70, 71]. Recently, opposing cascade regulation by BSL phosphatases in separate compartments (YDA^MKKK^ activation at plasma membrane, MPK6 deactivation in the nucleus) was shown to control stomatal fate [72].

Furthermore, plant receptor complexes are recruited to distinctive microdomains that could compartmentalize signals near the plasma membrane [73]. This resembles phase separation of human T cell receptor signaling which clusters molecules together upon activation and protects downstream kinases from phosphatase activities [74].

## Combinatorial signaling

**Fig. 5:**
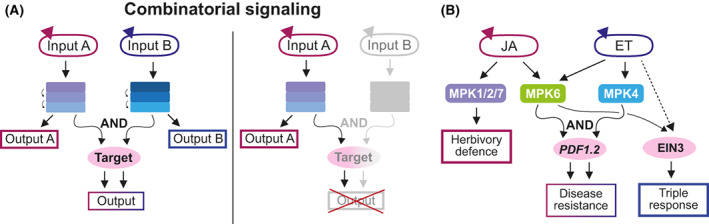
Combinatorial signaling. (A) When an output is specified by the combination of two independent signaling pathways, and activation of only one cannot lead to the output, such “AND” gate mechanism is called combinatorial signaling. (B) In *Arabidopsis*, ET (ethylene) and JA (Jasmonic acid) pathways demonstrate how combining signals from MPK4 and MPK6 may provide output specificity. Different combinations of common and unique MAPKs activated by ET and JA could contribute to both common and pathway‐specific outputs.

Combinatorial signaling occurs when the concomitant action of two or more independent signals are required to evoke a particular output, which effectively acts as a “molecular AND gate” [26] (Fig. 5A). Even if the two pathways activate a shared component, the co‐signaling with a unique component branching from one of the pathways would enhance specificity with such combinatorial signaling. Mammalian ERK^MAPK^ is a shared component central to signaling pathways that determine cell migration, division, and survival. To evoke the specific output of epithelial cell survival, ERK requires the combined inputs from growth factor signaling and cell adhesion [75]. The epidermal growth factor (EGF) ligands bind to its cell surface receptor to initiate intracellular Raf^MKKK^‐MEK^MKK^‐ERK^MAPK^ cascade, while the cell adhesion elicits regulators that could modulate cascade activation and nuclear translocation of ERK [75, 76].

In *Arabidopsis*, the induction of the defensin gene *PDF1.2* requires simultaneous activation of jasmonic acid (JA) and ethylene (ET) pathways [77, 78]. JA plays an important role in wounding response and defense against herbivory, while ET is a stress hormone highly responsive to various biotic/abiotic stimuli [79, 80]. *PDF1.2* expression thus serves as the “AND gate” that integrates two separate inputs from JA and ET: lacking either pathway would abolish the output (Fig. 5B). PDF1.2 defensin contributes to broad spectrum disease resistance and can be activated by fungal pathogen but not by wounding alone [81]. The shared component MPK6 is activated by both JA and ET signaling [78, 82], whereas the pathway‐specific component MPK4 is required for ET‐induced PDF1.2 activation but is not activated by JA [78, 83]; and these two branches converge for *PDF1.2* induction (Fig. 5B).

It was recently reported that JA signaling activates MKK3‐MPK1/2/7 with slow kinetics, which is independent from the rapid activation of MKK4/5‐MPK3/6 after wounding [84]. These findings contradict the MKK3‐MPK6 module activated by JA [78], we therefore add an additional branch of MPK1/2/7 under JA without eliminating MPK6 (Fig. 5B). This branch is important for JA‐induced defense against herbivory, as *mkk3* mutant shows enhanced susceptibility to a lepidopteran herbivore [84]. MPK4 is not required for all ET outputs, as upon treatment of ET precursor ACC, *mpk4* still exhibits the “triple response” in dark‐grown seedlings [83] typical of ET response [85, 86]. EIN3, a key transcription factor regulating ET‐induced genes, is indispensable for the triple response, and its stability controls ET outputs. EIN3 is phosphorylated by MPK6, which lead to increased stability [87, 88]. This model shows how combining the inputs from MPK4 and MPK6 downstream of ET and JA respectively provide specificity for a range of pathway outputs.

## Cross‐pathway inhibition

**Fig. 6:**
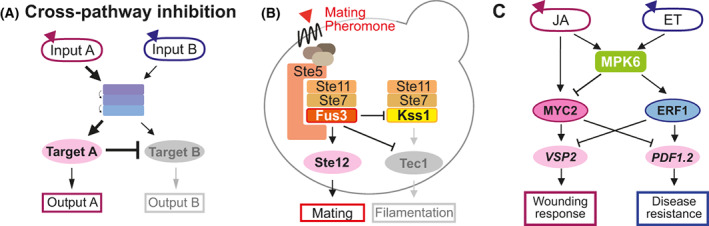
Cross‐pathway inhibition. (A) Cross‐pathway inhibition can improve output specificity even when two pathways share signaling components. The target of pathway A inhibits the target of pathway B. So, input A could prevent the activation of output B, ensuring a more specific output for pathway A. (B) In yeast, the mating specific Fus3^MAPK^ enhances mating output by suppressing Kss1^MAPK^ and the Kss1^MAPK^ target Tec1, which is required for filamentation. This improves output specificity even when Fus3^MAPK^ and Kss1^MAPK^ are both activated by Ste11^MKKK^ and Ste7^MKK^. (C) Bilateral inhibition of JA and ET pathways in *Arabidopsis* is shown by the opposing regulation of MYC2 and ERF1 on wounding and defense genes. Though MYC2 is mainly activated by JA signaling, MPK6 downstream of JA also suppresses MYC2 transcription.

When a downstream component of pathway A inhibits a downstream component of pathway B, it could diverge pathway outputs even with shared components: Input A only produces output A, as it prevents output B; and input B activates output B only when input A is absent (Fig. 6A). An example of this is the cross‐pathway inhibition between the mating pathway (Fus3^MAPK^) versus the invasive growth pathway (Kss1^MAPK^) in yeast. Though the mating pheromone could activate both Fus3 and Kss1, the mating specific Fus3 prevents Kss1 from activating Tec1 (a transcription regulator for invasive growth) by suppressing Kss1 activity [89] and by phosphorylating Tec1 to promote its degradation [90, 91] (Fig. 6B). Mutual insulation requires additional mechanisms (such as scaffolding) to help prevent invasive growth signaling from leaking into the mating pathway [16].

Bilateral inhibition of two pathways could improve mutual insulation. In Arabidopsis, downstream of ET signaling, MKK9‐MPK6 activates ERF1 [82], a transcription factor that promotes defense‐related genes such as *PDF1.2* and inhibits wounding responsive genes such as *VSP2* [92, 93]. Conversely, JA signaling activates MYC2, which promotes wounding responsive genes (e.g., *VSP2*) and inhibits defense‐related genes (e.g., *PDF1.2*) [78, 92]. This mutual inhibition improves ET/JA pathway insulation: with ET signaling via ERF1 upregulates defense‐related genes and JA induced‐MYC2 activates wounding responsive genes (Fig. 6C). But JA pathway also includes a feedback regulation with MPK6 suppressing MYC2 transcription [78]. In effect, although MPK6 is involved in both JA and ET signaling pathways, it plays a positive role for ET related‐defense gene activation but inhibits wounding responses via suppressing MYC2 (Fig. 6C). Mutual inhibition between MPK4 and MEKK2 leads to feedback loops [94, 95]. Disruption of MEKK1‐MKK1/2‐MPK4 releases the negative regulation on MEKK2, and activation of MEKK2 leads to SUMM2‐mediated immune response [96, 97]. This immune response further induces MEKK2 expression, which suppresses MPK4 activity thus further amplifying SUMM2 signaling [95].

## Fine‐tuning

Signaling networks often employ more than one insulating mechanism to improve specificity. Fine‐tuning of signaling activity at different levels could also contribute to specificity: modulation of the amplitude and/or duration of cascade inputs, regulation of activation and deactivation kinetics, positive and negative feedback loops.

Input modulation occurs at multiple levels such as ligand processing/diffusion and receptor complex assembly/stability. Recent findings show that mechanical signals modulate input intensity via regulating receptor expression in both plants and animals. Plant tissue damage increases pathogen‐pattern recognition receptor FLS2 expression to enable perception of damage‐inducing invaders [98], while soft media reduces EGF receptor expression and ligand binding dynamics in mammary epithelial cells [99].

Sustained versus transient signals of ERK^MAPK^ are sufficient to dictate the outcomes of differentiation versus proliferation in mammalian PC12 cells [100]. Sözen *et al*. [84] suggested that differential transcriptional regulation of the MKKK genes in Arabidopsis could confer signaling specificity. The authors showed that wounding and JA transcriptionally activates the fast‐responding MKKK14 (15–30 min), whereas MKKK17, 18, 19, 20 are induced by wounding much later (1–2 h) and to a lesser extent. Though the exact output difference is unclear (as they share MKKs‐MAPKs), the different MKKK induction patterns may exert strong and transient versus weak and sustained signals to fine‐tune outputs [84].

Activation versus deactivation rates of kinases control signal amplitude and duration. Many of the insulating mechanisms described here are conditioned by these rates. For example, compartmentalization can be further tuned by differential activation/deactivation rates in distinct compartments. ERK1/2^MAPKs^ are deactivated faster in the nucleus than the cytoplasm, thus requiring continuous shuttling of activated proteins into the nucleus. Indeed, the slower nuclear trafficking of ERK1 compared to ERK2 reduces its capacity to produce signaling output [52]. MAPK deactivation occurs rapidly and efficiently via dephosphorylation by phosphatases [40]. MAPK transcription and protein turnover must also contribute to their activation/deactivation kinetics, yet examples are scarce. Interestingly, MAPK phosphatases are often regulated by the MAPKs they dephosphorylate, forming a feedback mechanism [40].

Positive and negative feedback mechanisms are common in MAPK cascades to amplify or tightly control outputs. For example, the yeast scaffold Ste5 contains binding domains for both activation and downregulation of the target Fus3^MAPK^ [38], and activated Fus3 in turn phosphorylates Ste5 to negatively regulate signaling outputs in a feedback loop [101]. These data show that scaffolds could precisely tune the quantitative outputs of a pathway. In Arabidopsis, JA exerts both positive and negative regulation to fine‐tune the expression and activity of MYC2 (Fig. 6C) [78, 93, 102], possibly because it is central to JA's crosstalk with other hormonal pathways. An example of a positive feedback loop includes the MKKK3/5‐MKK4/5‐MPK3/6 cascade activated by bacterial flagellin peptide flg22: the activated MPK6 phosphorylates MKKK5 to further enhance the cascade signal [103]. The positive feedback loop formed between BASL and MPK3/6 in concert with the scaffolding activity of BASL establishes polarity in the cell cortex [48].

## Future perspectives for plant MAPK signaling

Plant MAPK cascade signaling shows more complexity compared to yeast and mammals, likely as an adaptation to the enormous expansion at receptor/ligand input level. The animal/yeast models appear more clearcut due to two reasons: (a) less crosstalk, (b) distinct/unambiguous readouts for outputs. In plants, many crosstalks are perhaps desirable. This is evident by the well‐known extensive interconnections among plant hormonal pathways [79] and large transcriptomic overlaps observed in both biotic and abiotic stress responses [104, 105, 106]. Reusing similar cellular signaling modules in multiple scenarios may be an efficient way to adapt to the fast‐changing environment.

The frequent crosstalks raise the challenge to distinguish common from unique outputs when assessing pathway specificity. Current readouts for pathway outputs in the plant field rely heavily on fast and highly responsive gene/protein markers and sometimes include signatures that are common among multiple pathways (e.g., ROS production, activation of MAPKs etc). It is often not known (not tested) if a particular pathway's marker could be as responsive if not more responsive to other inputs. Given the nature of complex signaling crosstalk in plants, many of these pathway markers are likely to respond to inputs other than the authentic ones. Furthermore, output assessments using collective functional traits (e.g., “triple response” in ET signaling [85], resistance/susceptibility to pathogens in defense signaling) may also need re‐evaluation, because these functional readouts might not represent the pure outcome of a specific input, but rather a combination of several associated pathways. This is especially true if the input is a phytohormone. The lack of cellular resolution additionally confounds the issue, as the “outputs” could be altered or simply coming from another pathway in a cell type different from the inputs. Recent advancement in methods such as single‐cell RNAseq could help improve this for the future. Functional studies in plants need to compare pathways in cell‐type‐specific manner and to isolate common from specific readouts. We believe that the establishment of unambiguous, single cell readouts is a crucial step toward understanding pathway crosstalk and specificity. For example, upon activation, a cell‐type‐specific transcription factor that is sufficient to mimic input stimulation of a pathway represents a clear readout.

Studying MAPK cascade signaling in plants has relied on functional analysis, typically gain or loss of function analyses. Given these multifunctional kinases often have discrete functions in different cell types, the major limitation is that constitutive global perturbation of these widely expressed, multifunctional proteins often lead to pleiotropic phenotypes (e.g., *yda* mutant), which can mislead functional interpretation. Furthermore, some members of MAPK cascade signaling are known to be guarded by the plant immune system, and thus perturbation of their function (e.g., *mpk4, mkk1/mkk2, mekk1* mutants) leads to autoimmune phenotypes (dwarfism or lethality) that mask their function in other processes [94, 107, 108]. Tissue‐specific manipulation of MAPK cascades signaling elements will help dissect their complex roles. For example, utilizing the constitutively active MKKs in stomatal cell lineages has helped the spatial–temporal dissection of cascade function during stomatal development [70, 71, 109]. With the emerging tool of tissue‐specific inducible CRISPR, it should be possible to obtain functional knock outs of MAPK cascade elements with cellular precision [110].

Furthermore, genetic redundancy observed for many MAPK cascades calls for a thorough investigation of all the members. For example, loss of function mutations of all *MKK1/2/3/7/9* are required for a defective phenotype in stigma receptivity [111]. The puzzling re‐occurrence of the same MAPKs (e.g., MPK3 and 6) downstream of many pathways while contributing different outputs may indicate extensive combinatorial signaling within the network. As many independent routes (MKKK‐MKK‐MAPK) can activate both the same MAPKs but also other distinct MAPKs, MPK3 and 6 in combination with different MAPKs could produce an array of different outputs. In addition, different routes may impose subtle differences such as in interacting partners, subcellular localization, and enzymatic activity to finetune the outputs. The involvement of the “other MAPKs” are often not known because of the total number of MAPKs and limitations in our current experimental systems to detect all their activities. Different rates of MAPK activation also contribute to this bias, as clade A MPK3 and 6 are fast responding compared to the slow responding clade C MPKs (1/2/7/14) that are less studied [84]. Live tracking of MAPK activity using FRET sensors [112] and development of single cell proteomics [113] may help us study specificity with a fuller network in mind.

Plant networks often employ more than one insulating mechanism assisted by various fine‐tuning regulations. This in turn creates many interlocking connections and nonlinear relationships that are difficult to understand intuitively. Mathematical modeling helps to simplify and better dissect such complex systems, as has been successful for Ca^2+^ wave decoding in plant communication with symbionts during root nodulation [114, 115], and to provide network prediction in complex developmental programs such as flowering decision and the spatial establishment of the shoot apical meristem [116, 117, 118]. The specificity models [26] reviewed here are general mechanisms that could theoretically apply to all other shared signaling elements such as Ca^2+^, ROS, and CDPKs, thus having broad implications on signaling specificity in plants. Modeling will become more powerful with the development of unambiguous pathway readouts with cellular precision. Together with tools to investigate the network on a global scale, we can make big steps in our understanding of plant signaling specificity.

**Glossary**

**ACC**: 1‐Aminocyclopropane‐1‐Carboxylic acid is an ethylene precursor.

**AGB1**: The G‐Protein β subunit (AGB1) belongs to the heterotrimeric G proteins complex in Arabidopsis, which comprise one Gα (GPA1), one Gβ (AGB1), and three Gγ subunits (AGG1, AGG2 and AGG3).

**ANPs**: Arabidopsis Nucleus and Phragmoplast localized protein kinases (including ANP1,2,3) belong to the MKKK family.

**At + Gene name**: Genes or proteins from *Arabidopsis thaliana* (At).

**BASL**: Breaking of Asymmetry in the Stomatal Lineage (BASL) is a polarity protein leading to the asymmetric division during stomatal formation.

**BSL phosphatases**: The BSU1 (*bri1* suppressor 1)‐Like (BSL) phosphatase family play key roles in stomatal development. Three BSL phosphatases (BSL1, BSL2, BLS3) directly interact with BASL to enable asymmetric cell division. At the plasma membrane, all four members of BSL contribute to positive regulation of YDA to promote stomatal differentiation, whereas in the nucleus, BSL2, BSL3 and BSU1 impose negative regulation on MPK6 that suppresses stomatal cell fate.

**CDPKs**: Calcium‐Dependent Protein Kinases play important roles including growth, development, stress responses and hormonal signalling in plants.

**Crosstalk**: Originally defined as unwanted signals in a communication channel caused by leakage from another circuit. In this review, it refers to the interferences between the pathways (signalling of one pathway can activate or suppress the signalling of another), it can be either desirable or undesirable.

**ERF1**: Ethylene response factor 1 is a bZIP transcription factor. Its promoter is targeted by the EIN3 (Ethylene insensitive 3) transcription factor.

**FAMA stage**: Three sequential stages of stomatal differentiation are defined by three different bHLH transcription factors: the SPCH stage initiates the transition of a cell to start division and stomatal lineage, the MUTE stage specifies guard mother cell, which will give rise to the guard cells, and the FAMA stage starts the final differentiation of the guard cells [119].

**Fus3**: Cell Fusion 3 is a yeast MAPK involved in mating decision. It is only activated during the mating pathway and remains inactive in other situations.

**GPT1**: Gluocose‐6‐Phosphate Translocator 1 is a gene important for lipid body accumulation during pollen development.

**Heterotrimeric G proteins**: Transmit diverse extracellular cues by coupling with the plasma membrane‐localized receptors and different signalling proteins inside the cells. It consists of three subunits: Gα, Gβ and Gγ.

**KSR1**: Kinase Suppressor of Ras‐1 is a mammalian scaffold protein. The Ras protein family belongs to the small GTPases.

**Kss1**: Kinase suppressor of Sst2 mutations‐1 is a yeast MAPK homologous to Fus3. It regulates filamentation and invasive growth.

**Mammalian MAPK families (see also Fig** **2** **B)**: ERK1/2 (Extracellular‐signal Regulated Kinase) pathways, also known as Ras‐Raf^MKKK^‐MEK^MKK^‐ERK^MAPK^, are involved in cell division and proliferation; p38 MAPKs, including p38α, p38β, p38γ and p38δ, are involved cell differentiation and apoptosis in response to stress stimuli; JNK (c‐Jun N‐terminal kinase) including JNK1/2/3, are also involved in stress responses, T cell differentiation and inflammation; ERK5 is specifically activated by MEK5^MKK^, and is involved in endothelial cell function.

**MAPK (or MPK)**: Mitogen‐Activated Protein Kinase. MPK is used when a specific MPK in plants is mentioned.

**MAPK cascade**: Commonly refers to the sequential phosphorylating cascades of MKKK‐MKK‐MAPK. This review does not mention MAP4Ks that act upstream of MKKKs. For easy recognition of cascade elements with noncanonical names, superscripts of MAPK, MKK or MKKK are used.

**MKK (or MAPKK, MAP2K, MEK)**: Mitogen‐activated protein Kinase Kinase.

**MKKK (or MAPKKK, MAP3K, MEKK)**: Mitogen‐activated protein Kinase Kinase Kinase.

**MYC2**: A bHLH transcription factor that acts as a master regulator of JA signalled plant immune responses.

**PDF1.2**: Plant Defensin 1.2 contributes to broad spectrum disease resistance and is ET and JA responsive.

**RACK1**: Receptor for Activated C‐Kinase 1 is a member of the WD40 repeat family of b‐propeller proteins. It was discovered through its ability to function as a scaffold protein, stabilizing signalling complexes involving protein kinase C.

**RLK/Pelle**: The plant Receptor Like Kinase with its kinase domain sharing homology to a cytoplasmic protein kinase (Pelle) that establishes dorsoventral polarity in Drosophila embryos. *Pelle* genes are also involved in immune response. Due to kinase homology, RLK/Pelle is also sometimes called IRAK (interleukin‐1 receptor‐associated kinase) which plays a central role in inflammatory responses in mammalian immune cells.

**ROS production**: Reactive Oxygen Species is a normal product of plant cellular metabolism and increased production is triggered by various environmental stresses.

**SCRM**: SCREAM is a basic helix‐loop‐helix (bHLH) transcription factor important for stomatal development. SCRM functions as a scaffold to bring MPK3/6 in proximity with SPEECHLESS (SPCH), thereby allowing SPCH phosphorylation and downregulation to inhibit stomatal cell fate.

**Ste5**: Sterility 5 is a yeast MAPK scaffold protein. It is recruited to the membrane upon induction of mating by appropriate mating pheromones.

**SUMM2**: A nucleotide‐binding leucine‐rich repeat (NLR) protein that guards the MKKK1‐MKK1/2‐MPK4 cascade, which triggers cell death when the cascade activity is disrupted.

**Triple response**: Typical ethylene response phenotypes observed in dark grown seedlings: shortening and thickening of hypocotyls and roots and exaggerated apical hook curvature.

**VIP1**: VirE2‐interacting protein 1 is a bZIP transcription factor.

**YDA**: YODA is a MKKK that functions for stomatal development, embryo development and immune responses.

**ZAR1**: Zygotic arrest 1 is a leucine‐rich repeat receptor‐like kinase that controls zygote elongation and asymmetric division via the ZAR1‐YDA‐MKK4/5‐MPK3/6 cascade.

## Conflict of interest

The authors declare no conflict of interest.

## Author contributions

YM conceived the idea. YM and JN wrote the manuscript and made figures.
